# Economic risk coding by single neurons in the orbitofrontal cortex

**DOI:** 10.1016/j.jphysparis.2014.06.002

**Published:** 2015

**Authors:** Martin O’Neill, Wolfram Schultz

**Affiliations:** Department of Physiology, Development, and Neuroscience, University of Cambridge, Downing Street, Cambridge CB2 3DY, UK

**Keywords:** Risk, Economic, Orbitofrontal cortex, Prediction error, Uncertainty

## Abstract

Risk is a ubiquitous feature of the environment for all organisms. Very few things in life are achieved with absolute certainty. Therefore, it is essential that organisms process risky information efficiently to promote adaptive behaviour and enhance survival. Here we outline a clear definition of economic risk derived from economic theory and focus on two experiments in which we have shown subpopulations of single neurons in the orbitofrontal cortex of rhesus macaques that code either economic risk per se or an error-related risk signal, namely a risk prediction error. These biological risk signals are essential for processing and updating risky information in the environment to contribute to efficient decision making and adaptive behaviour.

## Highlights

• Economic risk is coded by single neurons in the orbitofrontal cortex (OFC).
• Risk coding by OFC neurons is mostly separate from value and visuomotor coding.
• OFC neurons code either economic risk per se or risk prediction errors.
• The OFC signals are necessary for monitoring and updating risky reward information.

## Graphical abstract

**Figure:**
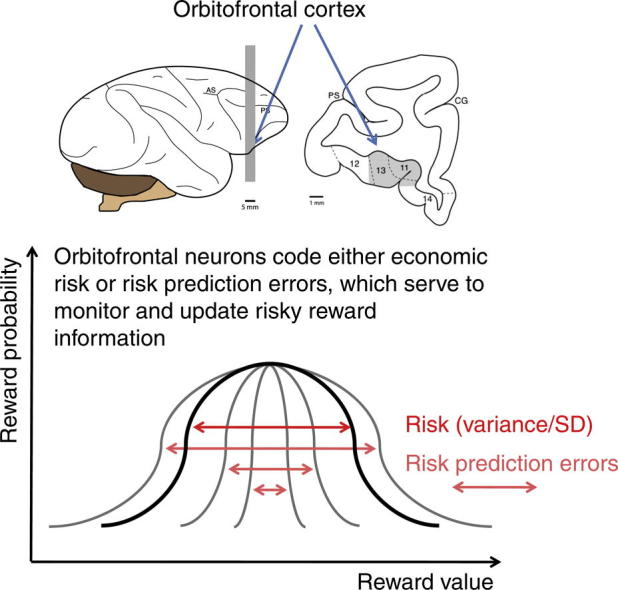

## 1 Risk in economic terms

Everyone is familiar with the concept of ‘risk’ but there is considerable variation in the common conception of the term, which can be easily exemplified by asking your friends what ‘risk’ means to them.

In 1921 the economist Frank Knight defined uncertainty as either ‘risk’ or ‘ambiguity’. Knight defined risk as a quantity with known outcome values and known outcome probabilities and ambiguity as a quantity with known outcome values and unknown probabilities. Essentially Knight defined ‘risk’ as a measurable quantity of uncertainty that is distinct from immeasurable uncertainty (ambiguity). Although this is an accepted definition of ‘risk’ in the field of economics, your friends will undoubtedly provide you with several other definitions of ‘risk’ as they understand it.

The variation in people’s definitions of risk is not simply due to naivety of the economic definition; the Oxford English Dictionary defines risk as ‘the possibility of loss, injury, or other adverse or unwelcome circumstance’. This dictionary definition invokes perhaps the most prevalent and common conception of risk outside of economic theory as a probabilistic sense of loss. This is not the case with the economic definition, as the possible outcomes in economic risk can be exclusively positive (or negative). Therefore, Knight’s contribution to the economic theory of risk was to define risk as a measurable quantity that captures the dispersion of probabilistic outcomes whether they are positive, negative or both.

**Fig. 1:**
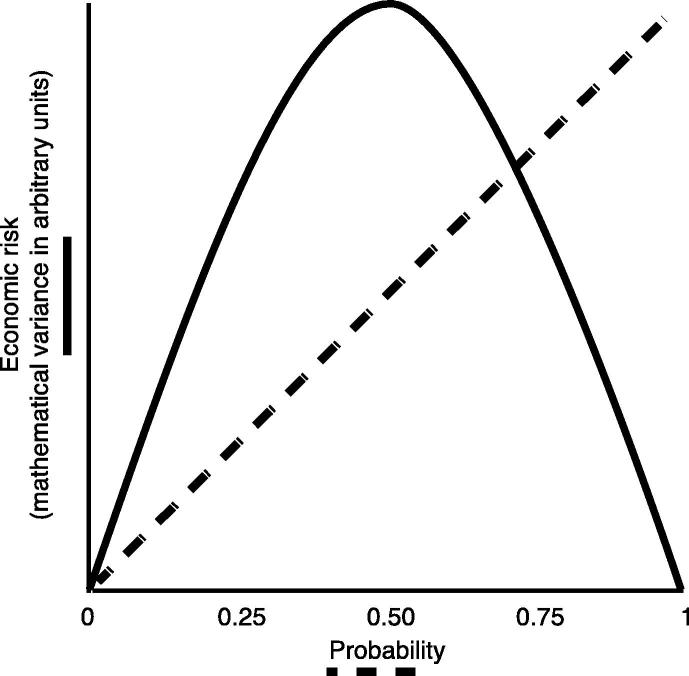
Economic risk and probability. Schematic of the non-monotonic relationship between economic risk, defined as mathematical variance, and probability. Economic risk is maximal when probability equals 0.5 and is minimal (zero) when probability equals 0 or 1.

The dispersion of probabilistic outcomes is typically measured as the variance, standard deviation (square root of variance) or the coefficient of variation (variance/expected value). Note that economic risk is distinct from reward probability, which has a non-monotonic relationship with risk: risk is maximal when probability is equal to 0.5 since the certainty that an outcome will occur increases from *p* = 0.5 to *p* = 1 and the certainty that an outcome will not occur increases from *p* = 0.5 to *p* = 0 (Fig 1; O’Neill and Kobayashi, 2009). As such, probability captures the likelihood of outcomes whereas risk captures the dispersion of possible outcomes (Burke and Tobler, 2011). For example, to demonstrate the distinction between economic risk and reward probability, imagine playing a simple game based on the outcome of tossing a coin. The probability of the coin landing on heads or tails is equal to 0.5. However, the economic risk in the game can be manipulated by offering different outcomes for heads or tails, say either 90p for heads or 10p for tails versus 60p for heads versus 40p for tails. The former option is considered more risky than the latter due to the larger dispersion in possible outcomes.

Note that in this example outcome probability = 0.5 due to the fact that there are two possible outcomes from a coin toss. The two-outcome approach has been utilised in the majority of neuroscientific investigations of risk processing, which comprise the focus of this article. This approach facilitates the most straightforward and simple method of orthogonalization of the independent variables of interest in such studies, namely reward probability, value and risk. Nonetheless, early work investigating the performance of patients with focal brain lesions using multiple outcomes highlighted the role of the ventromedial prefrontal cortex in risk processing (Sanfey et al., 2003), a finding that has subsequently been corroborated in task designs using the two-outcome approach (Xue et al., 2009). Therefore, risk may be processed similarly in tasks with either two outcomes or multiple outcomes.

A key area of interest for economists, psychologists and behavioural ecologists is whether organisms are sensitive to economic risk and the effect of economic risk on decision making and adaptive behaviour. Also, neuroscientists have begun addressing whether particular areas of the brain are responsible for processing economic risk, a putative necessity for informing decision-making mechanisms and adaptive behaviour.

### 1.1 Economic risk coding by single neurons in the orbitofrontal cortex (OFC)

Damage to the ventromedial prefrontal cortex (VMPFC) in humans has been shown to affect decision making under conditions of uncertainty in gambling tasks involving probabilistic judgements (Rogers et al., 1999; Clark et al., 2008) and economic risk (Sanfey et al., 2003). Activations in the human OFC, which is adjacent to the VMPFC, vary with economic risk (Critchley et al., 2001; Hsu et al., 2005; Tobler et al., 2007). Although these studies indicate a role of the OFC in risk processing, it is not possible to directly infer a role of single neurons in the OFC based on lesion studies or imaging techniques such as fMRI, which lacks the spatial and temporal resolution required. Specifically, lesion studies provide data that results from the absence of neurons and fMRI data captures the flow of oxygenated blood (BOLD signal) within voxels that contain several thousand neurons. Also, the temporal sampling of the BOLD signal is too low to detect brain activity at the frequencies of the firing rates of single neurons. Therefore, the detection of specific signals from individual neurons pertaining towards specific decision variables, such as risk and value, is not possible with current fMRI techniques. Therefore, we designed an experiment to test whether single neurons in the OFC process economic risk (O’Neill and Schultz, 2010). We employed rhesus macaques as an animal model and we designed a behavioural risk experiment for the monkeys to perform while we inserted single electrodes in the OFC and recorded from single neurons in the awake behaving monkey.

**Fig. 2:**
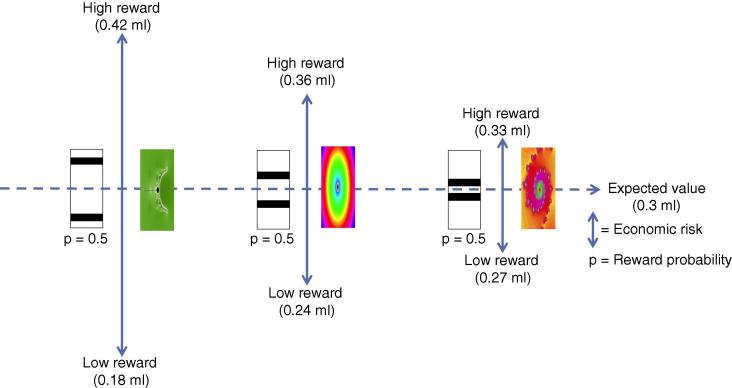
Risky cues. Monkeys were trained to associate visual cues with risky reward outcomes. The vertical position of the horizontal bars indicates the reward (volume of juice) to be delivered at the end of a trial: the higher the bar, the greater the volume of juice. Two bars indicate that one of two possible reward volumes would be delivered with a probability of 0.5. Monkeys were also trained on an additional set of fractal cues, with no explicit visual information, to control for any possible effects of the visual properties of the bar cues on behaviour and neuronal activity. Reward probability and expected value were equal for all cues.

Monkeys were trained to associate different visual cues with three binary symmetric equiprobable reward distributions that differed in economic risk (Fig 2). This design is essentially a simulation of the coin toss example described above: reward probability for all risk cues was equal to 0.5 and only the economic risk varied. Likewise, the expected value was equal for each risk cue. The orthogonal relationship between mean and variance facilitates manipulation of variance with fixed expected value. This is formally referred to as a mean preserving spread of risk (Rothschild and Stiglitz, 1970). This approach provides a framework in which sensitivity to risk can be experimentally tested in procedures that control for reward maximisation strategies and directly assess whether or not animals are sensitive to risk information per se.

Thus, this approach allowed us to behaviourally assess the monkeys’ risk preference and neuronal responses independent of a confounding reward maximisation strategy on behaviour or an expected value signal in neurons. In addition, we trained the monkeys on two sets of risky cues that varied in visual properties in order to control for potential neuronal responses coding the visual properties of the cues. The cues were presented on either the left or right side of a computer monitor requiring the monkeys’ to make eye movements to the corresponding side in order to receive reward to test whether neuronal responses were related to the action required to receive reward. Also, riskless cues that varied only in reward magnitude were used to explicitly test for any sensitivity of neurons to changes in value.

**Fig. 3:**
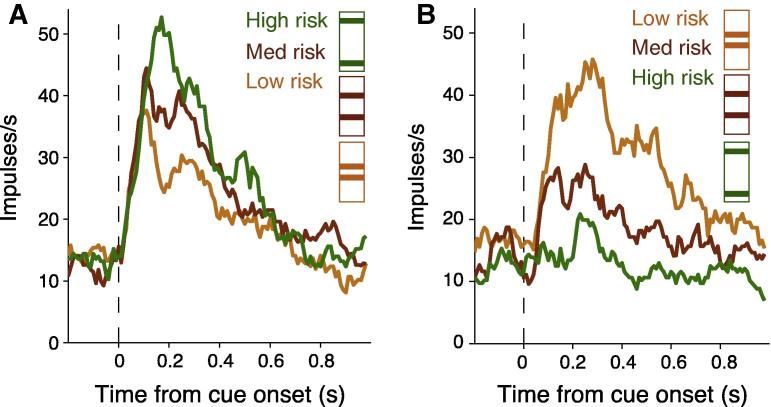
Orbitofrontal neurons code economic risk. Smoothed histograms show responses from two example neurons coding economic risk with positive slope (A) and negative slope (B). Color coding of the cues in the figure legend is for presentation purposes only. Data from O’Neill and Schultz (2010), with permission from Elsevier.

**Fig. 4:**
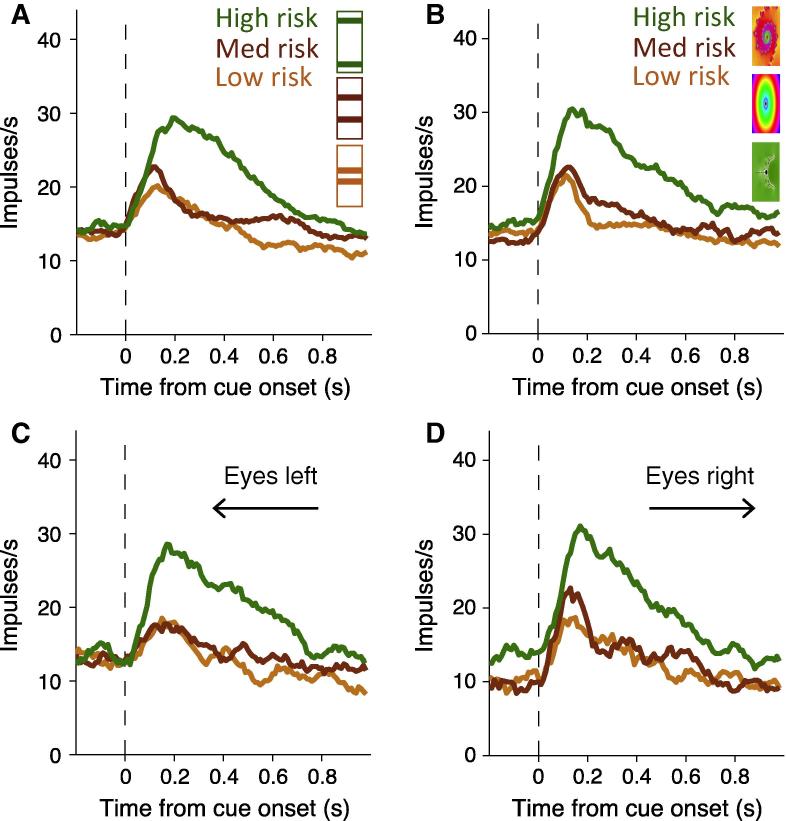
Orbitofrontal neurons code economic risk independent of visuomotor contingencies. Smoothed histograms show averaged population responses from neurons coding economic risk independent of whether it was indicated by bar cues (A) or fractal cues (B). The same population of neurons also coded economic risk independent of whether an eye movement to the left (C) or right (D) was required for reward. Color coding of the bar cues in the figure legend is for presentation purposes only. Data from O’Neill and Schultz (2010), with permission from Elsevier.

When the monkeys were given a choice between the risky cues and a safe cue of the same expected value they preferred the risky options with a monotonic increase in preference with higher risk. This preference was independent of the visual properties of the cues; the monkeys preferred the higher risk options irrespective of whether the risk information was explicitly displayed on the cues or not (bar and fractal cues in Fig 2, respectively). They also responded quicker to the more risky cues. Taken together these results suggest the monkeys were risk seeking. Recordings from single neurons revealed subpopulations of OFC neurons that were sensitive to the economic risk associated with the visual cues and represented the economic risk by an increase or decrease in firing rate (Fig 3A and B respectively). Fig. 4A and B shows OFC neurons coding risk independent of the visual properties of the cues. Fig. 4C and D shows the same OFC neurons coding risk independent of the spatial location of the cues and the eye movement required for reward. Therefore, the risk signal observed in OFC neurons is independent of visuomotor contingencies.

**Fig. 5:**
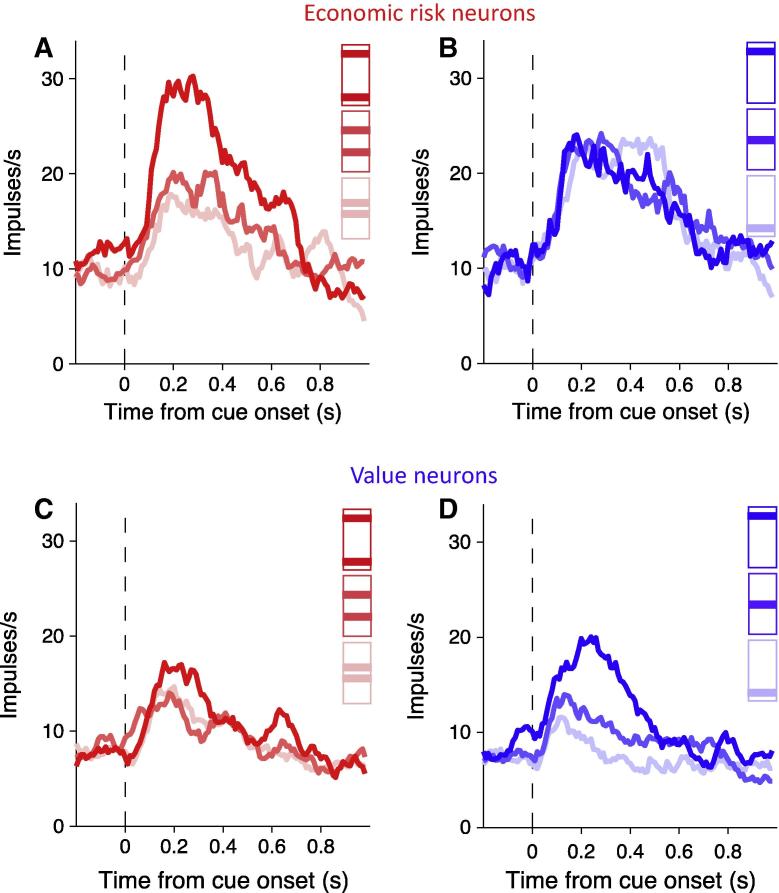
Economic risk and value coding. Smoothed histograms show orbitofrontal neurons that coded either the economic risk associated with reward and not value (A and B) or the value and not the risk (C and D). Color coding of the cues in the figure legend is for presentation purposes only. Data from O’Neill and Schultz (2010), with permission from Elsevier.

Since the monkeys preferred the riskier options it was imperative to test whether the neuronal responses were indeed reflecting the risk information conveyed by the cues or the monkeys behavioural preference. This was tested in a subpopulation of risk sensitive neurons by presenting the monkeys with safe value cues that varied only in value and not risk. The majority of neurons tested with both risk and value selectively responded differentially to either risk or value (Fig 5), albeit a small but statistically significant population also responded to both. Of the 126 cue-responsive neurons tested with both the risk and value cues, 16 (13%) correlated significantly with risk and 42 (33%) correlated with value. The separate coding of value and risk precludes salience coding in these neurons as both risk and value convey salience to a similar degree, as indicated in the animals behavioural preferences and reaction times, yet elicit differential neuronal responses.

This finding contrasts with a study suggesting salience coding by OFC neurons (Ogawa et al., 2013). However, in this study the neuronal responses also appear to reflect the animals’ behavioural performance. Therefore an alternative explanation for the observed findings may be that the OFC neurons are coding other aspects of behavioural performance independent of salience, specifically the animals’ subjective preference for the different reward conditions, which could be driving both the behaviour and the neuronal responses independent of salience. Given that we tested for this potential caveat by comparing the risk and value responses of OFC neurons it is unlikely that the neurons responding significantly to risk and not value in our study reflect the salience of the risk cues, as we would expect a similar neuronal response to the value cues if this were the case. In addition, a fMRI study in humans has identified a value signal in the OFC that is independent of salience coding (Kahnt et al., 2014). Nonetheless, a small but statistically significant proportion of neurons coded both risk and value (7/126; 6%) therefore displaying activity that could be driven by salience coding. Future studies of risk and salience coding will benefit from clear orthogonalization of behavioural measures, risk and salience in order to clearly delineate the effects of these variables on neuronal activity.

### 1.2 Economic risk prediction error coding by OFC neurons

In a follow up study to our identification of risk coding neurons in the OFC we identified an error-related signal in OFC neurons related to risk, namely a risk prediction error (O’Neill and Schultz, 2013).

Prediction errors are a general phenomenon that can be derived from any variable that can be measured and represents the discrepancy between what is *predicted* to happen and what *actually* happens. For example, whenever we expect anything, such as the temperature outside when we leave the house in the morning, the brightness of our computer monitor when we first turn it on or how much we are going to enjoy an article on the coding of economic risk by OFC neurons, we generate a prediction and any violation of this prediction is considered a prediction error. Numerically a prediction error is calculated as the experienced outcome minus the expected outcome.

**Fig. 6:**
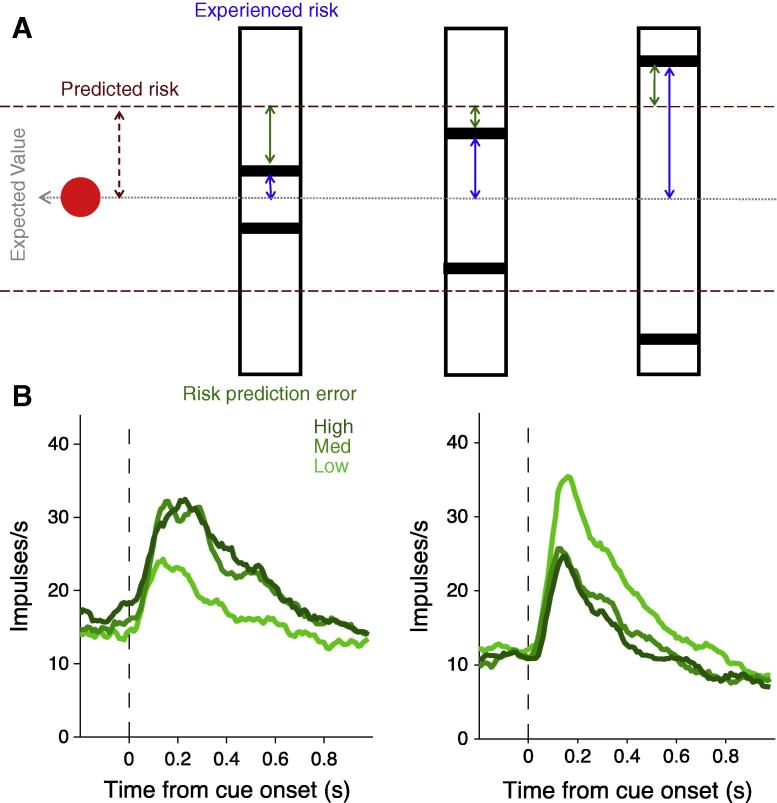
Orbitofrontal neurons code risk prediction error. (A) Schematic illustration of the measures of predicted risk, experienced risk and risk prediction error (experienced risk minus predicted risk) for each risky cue. (B) Smoothed histograms show averaged population responses from neurons coding risk prediction error with positive slope (left) or negative slope (right). Data from O’Neill and Schultz (2013).

Following this rationale, a risk prediction error is calculated as experienced risk minus predicted risk. In our experiment, the predicted risk is the risk measured as the standard deviation of the reward distribution of all possible outcomes in the experiment (six in total). The experienced risk is the risk indicated by the visual cue on any given trial. Therefore, there is a predicted level of risk at the beginning of a trial when the monkeys are required to fixate on a neutral stimulus (a red dot in the centre of the computer monitor). This predicted risk is calculated as the standard deviation of the six possible rewards available at the end of the trial (Fig 6A, maroon arrow). Subsequently, a risk cue appears presenting the experienced risk on a given trial, calculated as the standard deviation of binary reward distribution represented by each cue (Fig 6B, blue arrows). The risk prediction error is the numerical difference between the predicted risk and the experienced risk (Fig 6A, green arrows). Note that in our experimental design the expected value was held constant for all risk cues therefore there was no reward value prediction error at the cue because the mean reward value prediction was equal at the time of the fixation spot and the time of the cue (and indeed throughout the whole trial).

We identified and characterised OFC neurons that code the absolute value of risk prediction errors with either an increase or decrease in firing rate (Fig 6B and C). The regression slope for these neurons is significantly greater for risk prediction error compared to risk per se (average standardised regression coefficient, SRC, for neurons with positive slope = 0.29 versus 0.02 for risk, *F*_(1,__28)_ = 5.31, *p* = 0.03; average SRC for neurons with negative slope = −0.26 versus −0.01 for risk, *F*_(1,__34)_ = 48.36, *p* < 0.001; one way ANOVA). In addition, the amount of variance explained, measured as the coefficient of partial determination, was not correlated between risk prediction error and risk (Pearson’s *r* = −0.02, *p* = 0.9). Taken together these data show that the population of OFC neurons coding risk prediction error are largely separate from the OFC neurons coding risk per se.

Therefore, single OFC neurons code either economic risk per se, as measured by reward variance or standard deviation, or deviations in experienced risk from predicted risk, namely a risk prediction error. These two separate pieces of information pertaining to rewarding outcomes under conditions of risk and uncertainty are likely to be vital for tracking risky information in the environment and comprise part of the neuronal architecture necessary for efficient decision making and adaptive behaviour in risky situations.

## 2 Other single neuron studies on economic risk

A previous study on single neuron activity in the rat OFC identified a decision-related risk signal (Kepecs et al., 2008). OFC neurons fired more (or less) when decisions involved maximal uncertainty (*p* = 0.5) compared to decisions with more or less certainty. An economic risk signal has also been identified in midbrain dopamine neurons (Fiorillo et al., 2003), posterior cingulate cortex (McCoy and Platt, 2005), supplementary eye field (So and Stuphorn, 2010) and the anterodorsal septal region in rhesus macaques (Monosov and Hikosaka, 2013). These studies identified single neurons that were selective for economic risk independent of reward probability. Moreover, the risk signal in the septal region was specific to rewarding outcomes – the neurons did not respond to risky stimuli predicting negative, aversive outcomes.

The risk signal in OFC neurons in our study (Figs 4C and D; O’Neill and Schultz, 2010) and in Kepecs et al. (2008) is independent of the actions required to receive reward, whereas economic risk signals in cingulate cortex (McCoy and Platt, 2005) and the caudate nucleus (Yanike and Ferrera, 2014) provide information about the actions required to receive risky rewards. Subpopulations of supplementary eye field neurons code either economic risk independent of action or related to action (So and Stuphorn, 2010).Therefore, economic risk appears to be coded in the brain at the single neuron level and in a distributed fashion, with a high level abstraction of risk represented in OFC and action-dependent risk represented in the cingulate cortex, supplementary eye field and caudate nucleus.

In addition, the onset of the risk signal in OFC neurons occurs as soon as 100 ms after cue presentation. This latency is shorter than the risk-related responses in dopamine neurons, cingulate cortex and caudate nucleus. Specifically, the risk-related responses in dopamine neurons begins relatively late after cue presentation and ramps up gradually until reward delivery (Fiorillo et al., 2003). In the cingulate cortex and caudate nucleus, the risk activity was most clearly observed after movement had been initiated or reward delivered, respectively (McCoy and Platt, 2005; Yanike and Ferrera, 2014). In the supplementary eye field, risk responses unrelated to action occurred at shorter latencies than action-related risk responses (So and Stuphorn, 2010). Therefore, the risk sensitive OFC neurons may constitute an early component of a system that processes risky information, perhaps before it is transmitted to other brain areas such as the ventral tegmental region (dopamine neurons), supplementary eye field, cingulate cortex and caudate nucleus. This early response conceivably allows the OFC neurons to participate in detecting risk in decision situations involving uncertainty.

## 3 A note on variability in risk attitude

Although the risk seeking nature of the monkeys in our experiment may seem at odds with humans, who are generally considered risk averse, this is not the case. Risk attitude is a non-stable phenomenon with many reported cases of inter- and intra-species variability. For example, as famously described by Kahneman and Tversky, humans tend to be risk averse in the gain domain and risk seeking in the loss domain (Kahneman and Tversky, 1979; Tversky and Kahneman, 1992). A similar observation has been reported in New World monkeys (Chen et al., 2006; Lakshminarayanan et al., 2011). Moreover, when humans play for low-stake gambles, they are risk seeking, a phenomenon known as the ‘playing for peanuts’ effect (Prelec and Loewenstein, 1991; Weber and Chapman, 2005). Despite the fact that the volumes of juice used in our experiment were behaviourally discriminable, they were small in volume in respect to the overall volume of juice the monkey would be delivered in a day and over the course of a week (20 ml/kg daily ration; 24 h free access to water per week during the experiment). Therefore, with an average juice volume of 0.3 ml per trial, it is conceivable that the risk-seeking attitude of the monkeys in this experimental context may reflect the ‘playing for peanuts’ effect observed in humans. The observation of risk seeking behaviour in experimentally controlled rhesus macaques is not restricted to our experiment and has been observed both in other animals in our laboratory and in other laboratories (McCoy and Platt, 2005; Seo and Lee, 2009; So and Stuphorn, 2010; Fiorillo, 2011; Heilbronner et al., 2011; Lak et al., 2014; Strait et al., 2014; Xu and Kralik, 2014) with one exception reporting weak risk aversion (Yamada et al., 2013). However, in the latter study monkeys that were deemed less ‘thirsty’ showed reductions in risk aversion, consistent with the ‘playing for peanuts’ effect.

## 4 Implications of neuroscience research on economic risk

Identification of the existence of a biological substrate for economic risk coding has implications for the application of behavioural economic theory to the study of neuroscience. There are several different theoretical approaches in the field of economic theory that attempt to capture and describe decision making under conditions of uncertainty. The predominant theories in economics and finance are expected utility theory (von Neumann and Morgenstern, 1944), prospect theory (Kahneman and Tversky, 1979) and the mean–variance approach (Levy and Markowitz, 1979). Prospect theory is essentially an extension of expected utility theory and both these approaches can be considered as categorically distinct from a mean–variance approach.

This categorical distinction is derived from the fact that neither expected utility theory nor prospect theory rely on risky information per se whereas risk (as variance) is a central tenet of the mean–variance approach. Under the normative assumptions of expected utility and prospect theory, a non-linear value function is capable of capturing risk attitude independent of a requirement on risk per se. For example, the curvature of a utility function captures the risk attitude of an organism as either risk averse (with a concave, decelerating utility function) or risk seeking (with a convex, accelerating utility function). The same rationale may be applied to descriptive, process-based explanations of risk sensitivity that take into account behavioural observations, learning theory and cognitive processes (Kacelnik and Bateson, 1997): risk sensitivity might represent a skewed weighting of influence of unconditioned stimuli (rewarding outcomes) that bias an organisms preference for a risky (conditioned) stimulus. In other words, with binary-symmetric outcomes, as in our experiment, if an organism has a non-linear accelerating convex value function then that organism will place more value on the higher of the two rewards compared to the lower reward and therefore will prefer higher risk situations as a direct consequence (and vice versa for an organism with a non-linear decelerating concave function). Neither of these normative or descriptive approaches requires risk information per se at the time of uncertainty (before outcome) to be processed by a decision maker. It is the non-linearity of the utility function that determines how much more a decision maker gains by higher outcomes compared to losing from lower outcomes. Alternatively, the mean–variance approach assigns critical relevance to the first two moments of reward probability distributions, namely the expected value and the variance. Accordingly, under the assumptions of the mean–variance approach, a decision maker will be influenced directly by the expected value and the variance of possible outcomes under conditions of risk and uncertainty.

Although our data do not preclude the existence of neuronal mechanisms that comply with the assumptions of other economic models of decision making under risk and uncertainty, they do support the existence of a neuronal architecture involving the OFC that is explicitly sensitive to risk and expected value in accordance with the mean–variance approach.

## 5 Conclusions

Economic risk signals have been identified in single neurons and in distributed brain regions. The OFC risk signal is unique because it is independent of sensory and motor information therefore representing a high level abstraction of economic risk information. This high level abstraction of reward-related information processing in the OFC is not unprecedented. Economic values measured as subjective preferences for different reward types are also represented by a high level abstract coding mechanism in the OFC (Padoa-Schioppa and Assad, 2006; Padoa-Schioppa and Cai, 2011). Moreover, the abstract economic value signal in OFC is transformed into an action-related value signal in lateral prefrontal cortex (Cai and Padoa-Schioppa, 2014). Thus the economic risk and value signals in single neurons in the OFC represent a high level abstraction of reward-related information processing that is likely transmitted to other brain structures to guide decision making, action selection and adaptive behaviour under risk and uncertainty.

Characterisation of the risk signal at the single neuron level in the OFC has advanced the knowledge provided by human studies that indicated a possible function for the OFC in processing risky information (Critchley et al., 2001; Hsu et al., 2005; Tobler et al., 2007). In particular with the demonstration that single neurons code risk and risk prediction error with either positive or negative slope (Figs. 3 and 6 respectively), and that separate neurons code either risk per se or risk prediction error. Indeed, it may not be possible with the current levels of sensitivity of fMRI techniques to detect such differential signals, which could conceivably cancel each other out in the signal obtained with fMRI, or be processed too quickly for detection.

## 6 Future directions

The risk signal observed in single OFC neurons to date is largely derived from objective measures of risk, which serve as an independent variable in the analysis of neuronal activity. Interestingly, the risk-related activity of neurons in the anterodorsal septal region is not exclusively objective as these neurons only code risk information pertaining to rewarding outcomes and not aversive outcomes (Monosov and Hikosaka, 2013). Moreover, the risk signal observed in human OFC and dorsolateral prefrontal cortex is modulated by the risk preference of individual subjects (Tobler et al., 2007, 2009). Therefore, in order to further elucidate the risk coding properties of neurons in the OFC, future studies will benefit from designs incorporating rewarding and aversive outcomes as well as manipulations of individual risk attitude to test how these factors affect the risk coding properties of these neurons.
